# Advances in Nanozyme Catalysis for Food Safety Detection: A Comprehensive Review on Progress and Challenges

**DOI:** 10.3390/foods14152580

**Published:** 2025-07-23

**Authors:** Renqing Yang, Zeyan Liu, Haili Chen, Xinai Zhang, Qing Sun, Hany S. El-Mesery, Wenjie Lu, Xiaoli Dai, Rongjin Xu

**Affiliations:** 1 School of Food and Biological Engineering, Jiangsu University, Zhenjiang 212013, China; 2 Faculty of Agricultural Engineering, Jiangsu University, Zhenjiang 212013, China; 3 School of Energy and Power Engineering, Jiangsu University, Zhenjiang 212013, China; elmesiry@ujs.edu.cn (H.S.E.-M.); lwj@ujs.edu.cn (W.L.);

**Keywords:** nanozymes, biomimic catalysis, sensing application, food safety monitoring

## Abstract

The prosperity of enzyme-mimicking catalysis has promoted the development of nanozymes with diversified activities, mainly including catalase-like, oxidase-like, peroxidase-like, and superoxide dismutase-like characteristics. Thus far, the reported nanozymes can be roughly divided into five categories, comprising noble metals, metal oxides, carbon-based nanostructures, metal–organic frameworks, and covalent organic frameworks. This review systematically summarizes the research progress of nanozymes for improving catalytic activity toward sensing applications in food safety monitoring. Specifically, we highlight the unique advantages of nanozymes in enhancing the performance of colorimetric, fluorescence, and electrochemical sensors, which are crucial for detecting various food contaminants. Moreover, this review addresses the challenges faced in food safety detection, such as the need for high sensitivity, selectivity, and stability under complex food matrices. Nanozymes offer promising solutions by providing robust catalytic activity, adjustable enzyme-like properties, and excellent stability, even in harsh environments. However, practical implementation challenges remain, including the need for a deeper understanding of nanozyme catalytic mechanisms, improving substrate selectivity, and ensuring long-term stability and large-scale production. By focusing on these aspects, this review aims to provide a comprehensive overview of the current state of nanozyme-based sensors for food safety detection and to inspire future research directions.

## 1. Introduction

Natural enzymes, typically proteins, exhibit high specificity and catalytic efficiency and thus play important roles in various fields [1,2,3]. However, their stability and activity can be limited by factors such as temperature [4,5,6], pH [7,8,9,10], and the presence of inhibitors [11,12,13], consequently hindering their widespread application in sensing analysis [4,5,6,7,8,9,10,11,12,13,14,15,16]. Therefore, the development of new artificial enzymes to replace natural enzymes is of great significance in expanding their sensing analysis [14,17,18]. With the rapid development of nanotechnology [19,20,21,22,23], multiple nanomaterials with enzyme-mimicking catalytic activity, named as “nanozyme” [14,18,24], have been successfully developed for use as an artificial enzyme [18,25,26,27].

**Figure 1:**
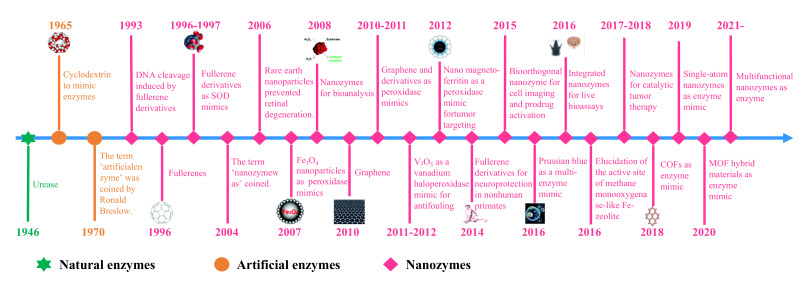
The timeline for the development of nanozymes [40].

Back in 2004, Manea proposed the professional term “nanozyme”, who applied organic ligands to modify gold nanoparticles (AuNPs), and then complexed with Zn^2+^ to prepare nanostructures with ribonuclease-like activity for cleaving phosphate esters [28]. Since the discovery of magnetic Fe_3_O_4_ nanoparticles with similar functions as horseradish peroxidase in 2007 [29], the development of nanostructures with natural enzyme-like properties has emerged as a pivotal field [30,31]. In 2013, Wei’s group summarized the nanomaterials with catalytic activity and also gave a specific definition of nanozymes. The development timeline of nanozymes is shown in Figure 1. Thus far, it is found that nanozymes exhibit diversified enzyme-like characteristics, mainly including catalase, oxidase, peroxidase, superoxide dismutase, etc. [32,33,34,35]. According to the structural compositions, the reported nanozymes can be roughly divided into noble metals (e.g., Au, Ag, Pt, Pd), metal oxides (e.g., Fe_3_O_4_, CeO_2_, ZnO, CuO), carbon-based nanostructures (e.g., carbon nanotubes, graphene oxide, and carbon dots), metal–organic frameworks (MOFs), and covalent–organic frameworks (COFs) [36,37,38,39].

When compared with natural enzymes [41,42,43,44], nanozymes with biomimic catalytic activities have outstanding advantages as shown in Table 1. First, nanozymes as nanostructures are endowed with the unique physical and chemical properties [45,46,47], such as the adjustable morphology, structure, and composition; Second, nanozymes are endowed with high structural stability, strong biocompatibility, and exhibit high biocatalytic a1ctivity under harsh surroundings [31,48,49]; Third, the unique merits of nanostructures with magnetic and photothermal effects, extend them with a wider range of application fields [49,50,51]; Four, the synthetic modes are simple and cost-effective and provide the possibility for large-scale production [22,52,53]. Owing to their excellent performance, nanozymes have been widely applied in several fields [54,55], becoming a hot research topic in the fields of chemistry and materials.

This review systematically summarizes the research progress of nanozymes toward sensing applications. Specifically, the classification and regulation strategies of nanozymes for enhancing their catalytic activity were introduced in detail, followed by a focus on their applications in sensing toward food safety, medical testing, and environmental monitoring fields. Lastly, this review discusses and looks forward to the challenges and future prospects in this field, aiming to provide reference and inspiration for the research and application of nanozymes, and further promote their application.

## 2. Background and Significance of Food Safety Detection

Food safety detection refers to the monitoring and analysis of various harmful substances in food to ensure it meets quality and safety standards. Key targets for detection include pesticide residues, heavy metals, mycotoxins, foodborne pathogens, and other harmful compounds. These contaminants can pose serious health risks to consumers, ranging from acute poisoning to long-term adverse effects such as cancer, neurological disorders, and reproductive problems.

Natural enzymes have been widely used in food safety detection due to their high catalytic efficiency and specificity. They can recognize and react with target analytes, enabling the detection of specific harmful substances. However, natural enzymes have limitations such as instability under harsh conditions (high temperature, extreme pH, etc.), short lifespan, and high production costs, which restrict their practical application.

Nanozymes, with their enzyme-mimicking catalytic properties, have emerged as promising alternatives to natural enzymes. They can provide comparable or even superior catalytic efficiency in food safety detection while offering enhanced stability, durability, and cost-effectiveness. In this review, we will compare the application principles and advantages of natural enzymes and nanozymes in the field of food safety detection, highlighting the potential of nanozymes to overcome the limitations of natural enzymes and offering new opportunities for advancing food safety monitoring technologies.

## 3. Fundamental Principles of Nanozymes

**Figure 2:**
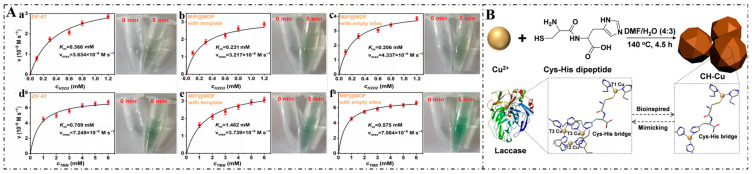
Catalytic kinetic activity of MOFs toward substrates: (**A**) Schematic diagram (Catalytic kinetic activity of ZiF-67 (a,d), unwashed MIP@MOF (b,e), washed MIP@MOF (c,f) as the substrates) [70] and (**B**) Mechanism diagram [69].

Nanozyme technology is rooted in the fundamental principles of enzymatic mechanisms, which involve the catalytic conversion of substrates into products through specific biochemical reactions [25,62,63]. The interaction between nanozymes and substrates can be explained through various theoretical models. For instance, the Michaelis–Menten model, which describes the rate of enzymatic reactions, can also be applied to nanozymes. The catalytic efficiency of nanozymes is influenced by their surface area, morphology, and the nature of the used nanomaterials [64,65,66]. These factors can enhance enzyme activity by providing a greater active site exposure and facilitating substrate binding [66,67,68]. For example, Zhang et al. demonstrated that functionalized MOFs exhibited remarkable peroxidase-like activity (Figure 2), which is attributed to their high surface area and stability across a wide range of temperatures and pH levels. Moreover, molecular docking simulation, a computational technique that predicts non-covalent host–guest interactions, is applied to elucidate the recognition mechanism of MOFs toward substrates at the molecular level [69,70].

Nanozymes play a pivotal role in improving enzyme activity and stability [51,71]. Their unique physical and chemical properties, such as high surface-to-volume ratios and tunable surface functionalities, allow for enhanced enzyme–substrate interactions. Liu et al. introduced an electric field-induced preconcentration that significantly improved the electrochemical detection of metal ions by enhancing the recognition of specific complexes. This method effectively reduced interference from non-specific ions, thus verifying the potential of nanozymes in enhancing sensing performance [72,73,74].

Moreover, the use of nanomaterials in nanozymes facilitates the development of advanced sensing technologies. The integration of nanozymes into electrochemical sensors has led to improved detection limit and faster response time [75,76]. For example, the incorporation of oxalate decarboxylase (OXDC) in sensing platforms has shown promise due to its metal-dependent catalytic activity and potential applications in clinical diagnostics and food processing [77]. The modulation of redox properties further illustrates the function of nanomaterials in enhancing enzymatic function [78].

## 4. Classification and Properties of Nanozymes

The synthesis methods of nanozymes are diverse, with each approach exhibiting distinct principles and applicability. Physical synthesis methods primarily include mechanical grinding and physical deposition, which employ physical means to break down raw materials or deposit them into nanoscale particles. These methods are suitable for processing heat-sensitive or poorly soluble materials, but they may encounter issues such as particle agglomeration and insufficient precision in size control. Chemical synthesis methods utilize chemical reactions in solution to generate nanoparticles, encompassing techniques like precipitation, sol-gel, and hydrothermal/solvothermal processes. These approaches enable precise regulation of nanozyme composition, size, and morphology, though they may require toxic reagents and stringent reaction conditions. Biological synthesis methods leverage biological entities or their extracts as reaction media, such as utilizing microbial metabolic processes or biomolecules in plant extracts to reduce and stabilize metal ions, forming nanoparticles. This method operates under mild, environmentally friendly conditions but suffers from low synthesis efficiency and suboptimal product purity. Additionally, template-assisted synthesis employs pre-constructed templates (e.g., DNA, MOFs) to guide nanostructure formation, enabling precise construction of complex nanostructures, albeit with increased process complexity due to template preparation and removal. Green synthesis strictly adheres to green chemistry principles, utilizing renewable resources and non-toxic solvents to synthesize nanozymes through simple, efficient, and low-cost methods, thereby reducing environmental footprints. The selection of synthesis methods must comprehensively consider factors such as target nanozyme type, desired performance, production scale, and cost-effectiveness to achieve efficient application in fields like food safety detection. Next, we will focus on the synthesis and characteristics of five categories of nanozymes: noble metals, metal oxides, carbon-based nanostructures, metal–organic frameworks, and covalent organic frameworks.

### 4.1. Noble Metals

Noble metal-based nanozymes have garnered significant attention due to their unique catalytic properties and widespread applications in sensing technologies [79,80,81]. These nanozymes can be classified primarily into two categories: metallic nanoparticles and alloy nanoparticles, both exhibiting distinct synthesis methods, properties, and applications [82,83,84].

#### 4.1.1. Synthesis Methods

The synthesis of noble metal nanoframes generally involves the use of sacrificial templates to assist or guide the formation of framework structures [85,86,87]. These templates are usually pre-fabricated using different protocols. There are five main methods of synthesis.

**Figure 3:**
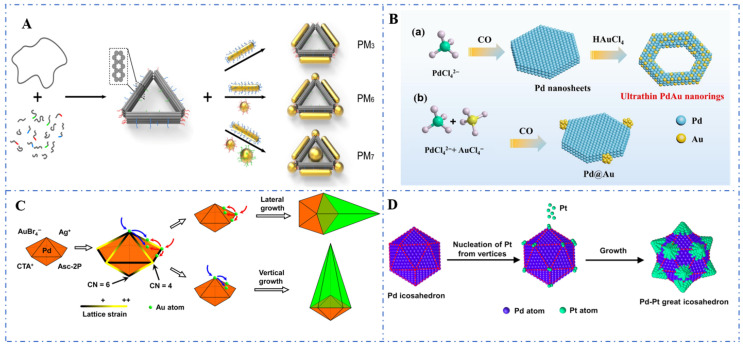
(**A**) Gold nanoparticle assembly guided by DNA origami templates [89]; (**B**) Etching to synthesize Pt nanocubes: (a) Schematic illustration for the growth process of PdAuNRs and (b) the compared route for other PdAu nanostructure [90]; (**C**) Selective deposition to form Pd-Au bimetallic nanocrystals [91]; (**D**) Electrochemical directional opportunity to form three-dimensional rhodium nanoframeworks [93].

(1) The first method is template-assisted assembly of nanoscale building blocks. This method involves using pre-fabricated templates to guide the spatial arrangement of nanoscale components [88]. For example, Liu et al. (2017) demonstrated the synthesis of gold nanoframes by assembling gold nanoparticles onto DNA Origami (Figure 3A), followed by the removal of the templates. This approach enables precise control over the nanoframe architecture through the design of the sacrificial template [89]. (2) The second method is facet-selective etching of solid nanocrystals. Guo et al. (2022) utilized facet-selective etching to transform solid platinum nanocubes into nanoframes. By selectively dissolving the facets using an oxidative etchant, they achieved porous platinum nanoframes with enhanced catalytic activity for oxygen reduction reactions (Figure 3B). This method leverages crystallographic anisotropy to create well-defined framework structures [90]. (3) Another method is a synthesis approach based on a seed-mediated growth strategy. Zhou et al. (2021) used Pd decahedra with fivefold twinning as seeds and achieved asymmetric growth of Au on the Pd seeds by controlling the slow reduction kinetics of the Au precursor (AuBr_4_^−^ instead of AuCl_4_^−^) and the weak reducing agent (ascorbic acid-2-phosphate, Asc-2P) (Figure 3C). This ultimately led to the synthesis of Pd-Au asymmetric nanopyramids [91]. (4) Then, another method is the dealloying of hollow alloy nanocrystals. Huang et al. (2023) developed platinum–nickel (Pt-Ni) nanoframes through the dealloying of hollow PtNi alloy precursors. By leaching away the less noble nickel component, they obtained porous Pt-rich nanoframes with high surface area, ideal for electrocatalytic applications. Dealloying offers a scalable route to tune composition and porosity simultaneously [92]. (5) The last method is the oriented deposition of nanoframes. Liu et al. (2021) reported the synthesis of rhodium nanoframes via electrochemical deposition guided by surfactants (Figure 3D). By controlling the growth direction of rhodium atoms on cubic templates, they achieved three-dimensional nanoframes with tailored geometries. This method highlights the role of kinetic control in framework formation [93].

#### 4.1.2. Properties

Noble metal nanozymes are known for their versatile catalytic and optical properties, making them highly valuable for various applications [94,95,96,97]. In photocatalytic systems, noble metals such as Pt, Au, and Ru significantly enhance charge separation and electron transfer dynamics when integrated with semiconductors like cadmium sulfide (CdS).

For instance, Pt-decorated CdS hollow spheres exhibit superior photocatalytic hydrogen evolution due to a higher Schottky barrier, which minimizes electron backflow and maximizes interfacial electron transfer efficiency. Au NPs also play a crucial role in enhancing the photocatalytic performance of CdS by facilitating electron transfer and reducing recombination rates [98]. In addition to their role in photocatalysis, noble metal NPs such as Au have been found to exhibit glucose oxidase (GOD)-like activity, catalyzing glucose oxidation through a two-step dehydrogenation mechanism followed by oxygen reduction to H_2_O_2_, similar to natural enzymes [99]. This GOD-like activity of Au NPs has been leveraged for the development of biosensors and bioanalytical tools. However, other noble metals like Pt and palladium (Pd) tend to favor the 4-electron oxygen reduction pathway to H_2_O, limiting H_2_O_2_ generation due to their catalase-like activity [100]. This difference in catalytic behavior highlights the importance of selecting the appropriate noble metal for specific applications. Moreover, the integration of noble metal NPs with flexible substrates has led to the development of portable and sensitive SERS chips. For instance, a flexible and stable SERS chip was fabricated by embedding Au@Ag NPs between an adhesive acrylic polymer tape and a polyethylene terephthalate (PET) film, enabling nondestructive detection of thiram on fruit peels [101]. This approach combines the advantages of flexible substrates and noble metal NPs, providing a promising tool for on-site food safety analysis [102].

### 4.2. Metal Oxides

Metal oxides have been developed as nanozymes due to their non-toxicity, simple preparation, good mobility, and relatively low cost [103,104,105]. These include magnetic Fe_3_O_4_ nanoparticles, copper oxide (CuO), cuprous oxide (Cu_2_O), cerium dioxide (CeO_2_), vanadium pentoxide (V_2_O_5_), manganese dioxide (MnO_2_), cobalt tetraoxide (Co_3_O_4_), and nickel oxide (NiO) [106,107]. It has been reported that these metal oxide nanozymes also exhibit simulated enzyme activities such as peroxidase, catalase, superoxide dismutase, and oxidase.

#### 4.2.1. Synthesis Methods

**Figure 4:**
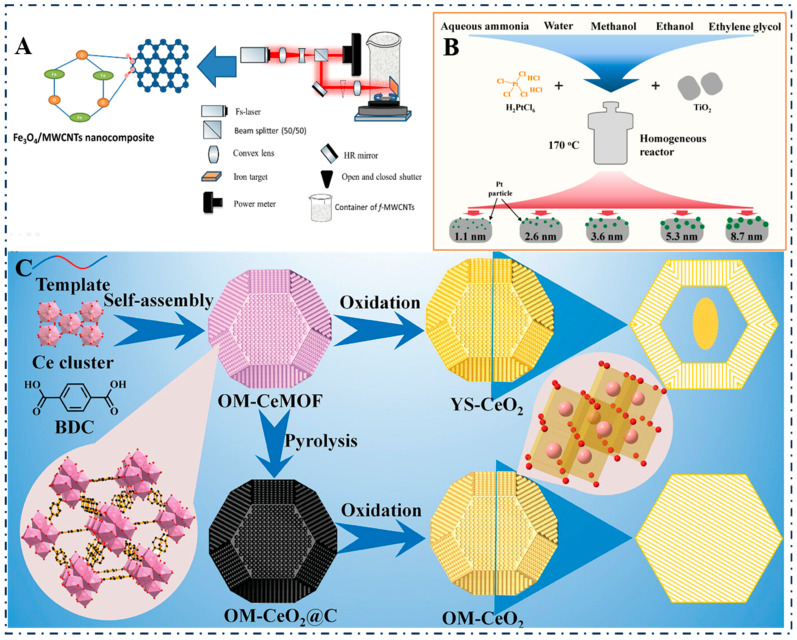
(**A**) Synthesis of Fe_3_O_4_ nanoparticles by pulsed laser ablation [111]; (**B**) Synthesis of Pt/TiO_2_ nanoparticles by hydrothermal/solvothermal method [113]; (**C**) Synthesis of OM-CeO_2_@C nanozymes by pyrolysis of MOFs precursors [114].

The synthesis of metal oxide nanozymes is mainly carried out through physical, chemical, and composite synthesis methods [108,109,110]. For example, Alkallas et al. (2023) used pulsed laser ablation to synthesize Fe_3_O_4_ NPs particles (Figure 4A), which exhibited good dispersibility and stability [111,112]. Meanwhile, Lei et al. (2024) employed hydrothermal/solvothermal methods to synthesize Pt/TiO_2_ nanoparticles (Figure 4B), precisely controlling the size, morphology, and purity of the nanozymes. The resulting products were relatively uniform and of high purity [113]. Furthermore, Chen et al. (2023) used MOFs as precursors, converting MOFs into metal oxide nanozymes through pyrolysis, thereby preparing high specific surface area OM-CeO_2_@C (Figure 4C) and enhancing their catalytic performance [114].

#### 4.2.2. Properties

Metal oxide nanozymes have shown broad application prospects in the fields of biomedicine and environmental detection due to their unique catalytic activity, high stability, and biocompatibility [115]. For example, iron oxide nanozymes (IONzymes) can decompose hydrogen peroxide to produce reactive oxygen species (ROS) under acidic conditions by simulating peroxidase activity, thereby effectively inhibiting the growth of intracellular Salmonella and promoting the autophagy pathway [116]. In addition, nanozymes such as MnO_2_ and CeO_2_ exhibit oxidase-like activity and can efficiently catalyze the oxidation reaction of colorimetric substrates (such as TMB), providing a sensitive colorimetric sensing platform for the rapid detection of organophosphorus pesticides (such as dichlorvos) [117]. The catalytic kinetic parameters of these nanozymes (such as the low Michaelis constant Km) indicate their high affinity for substrates, further supporting their application in complex biological environments [118]. Moreover, the multivalent characteristics of metal oxide nanozymes (such as Fe^2+^/Fe^3+^, Mn^2+^/Mn^3+^) and surface modification strategies (such as functionalized ligands or composite materials) can significantly enhance their catalytic efficiency and selectivity, laying the foundation for the development of multifunctional nano-diagnostic and therapeutic systems [119].

### 4.3. Carbon-Based Nanostructures

Carbon-based nanomaterials have also been found to possess enzyme-like activities, such as carbon dots, graphene oxide, carbon nanotubes, and fullerenes [64,120,121,122]. Their enzyme-like activities are attributed to the surface groups and unique electronic structures of carbon-based materials [123,124,125]. Therefore, the activity of carbon-based nanozymes can be greatly improved through rational design. These tunable carbon-based nanozymes have attracted widespread attention from researchers due to their excellent physical and chemical properties [126,127,128].

#### 4.3.1. Synthesis Methods

Carbon dots can be synthesized by different synthesis routes like microwave-assisted, hydrothermal synthesis, arc discharge, laser ablation, etc. [121,129,130,131,132]. Zhang et al. (2024) employ citric acid and m-phenylenediamine to synthesize N, P-codoped carbon dots (N, P-CDs) by a microwave-assisted method. Anhydrous ethanol and phosphoric acid are essential to the properties of N, P-CDs [133,134]. Carbon nanotubes can be prepared by plasma-based methods such as arc discharge and laser ablation, thermal preparation methods like chemical vapor deposition, and hydrothermal methods [135,136,137]. Shao et al. (2023) developed a field-effect transistor (FET) biosensor based on semiconductor-enriched single-walled carbon nanotubes (sc-SWCNTs) functionalized with norfentanyl antibodies for the sensitive detection of norfentanyl using the method of electrodeposition [138]. Fullerenes are commonly prepared by the arc discharge method. The operation is carried out in a high-temperature furnace. Carbon clusters are annealed with the help of a buffer gas passing through the quartz tube [139,140,141]. The temperature of the tube is maintained between 25 and 1000 °C. The reaction results in the consumption of the carbon electrode at the negative terminal.

Fullerenes are obtained by the annealing process of carbon after it has condensed. They are then collected in the water-cooler trap. C60 can be separated from the mixture using chromatography. Graphene oxide is commonly synthesized using the modified Hummers’ method [142]. Graphite in its flaky or powdery form is added to a protonated solvent (solution of sulfuric acid and sodium nitrate) [143]. The mixture is then reacted with H_2_O_2_ to eliminate metal ion impurities. Consequently, the color of the solution changes from dark brown to yellow. Graphene is obtained after centrifuging, washing, and freeze-drying the solution [144,145].

#### 4.3.2. Properties

The core properties of carbon-based nanostructures are determined by their atomic arrangement and dimensional characteristics [146,147]. Recent research has further revealed their unique behaviors in electrical, mechanical, optical, and chemical aspects. In terms of electrical properties, graphene has a high room-temperature carrier mobility [148]. For single-walled carbon nanotubes, the semiconductor purity has been improved through catalyst engineering, and their bandgap is linearly related to the inverse of the diameter [149]. In terms of mechanical properties, the compressive modulus of three-dimensional graphene foam is very high, and it can fully recover after being strained [150]. In the optical field, the fluorescence quantum yield of the content of carbon quantum dots is very high due to surface passivation, making them suitable for the nanozyme field [151]. In terms of chemical properties, the carboxyl density of graphene oxide can enhance its dispersibility in composite materials [152]. The precise control of these properties provides a basis for the design of multifunctional devices.

### 4.4. Metal-Organic Frameworks

Metal–organic frameworks (MOFs) are crystalline hybrid materials composed of inorganic metal nodes combined with suitable organic linkers [64,153,154], characterized by their highly ordered structural arrangement [17,155,156]. They exhibit high specific surface area, excellent stability, and flexible nanoscale porosity. Due to the presence of abundant active sites and transition metals, many MOF materials have been reported to possess enzyme-mimicking catalytic activity [157]. Owing to their superior enzyme-mimicking catalytic activity and high substrate affinity, MOF-based nanozymes have been widely applied in the field of sensing [158,159,160].

#### 4.4.1. Synthesis Methods

**Figure 5:**
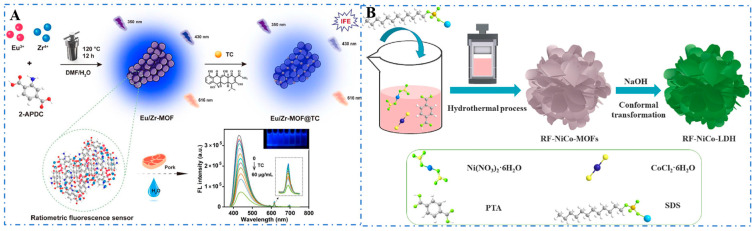
(**A**) Eu/Zr-MOF@TC [163] and (**B**) RF-Nico-LDH [164] synthesized by the hydrothermal method.

Most MOFs are synthesized via hydrothermal or solvothermal methods (Figure 5). Solvothermal methods are favorable for the slow crystallization of MOFs, yielding single crystals or highly crystalline powders that are suitable for structural analysis [161,162]. Zhang et al. (2024) developed a novel ratiometric fluorescence sensor based on a bimetallic metal–organic framework (Eu/Zr-MOF) via hydrothermal synthesis for the detection of tetracycline [163]. Meanwhile, Tang et al. (2024) prepared rose-like NiCo-LDH derived from bimetallic NiCo-MOF and constructed a sensing platform with a large electrocatalytic surface area, high conductivity, and good stability using N_2_H_4_ [164]. Recently, many studies have employed MOF-on-MOF heterostructures composed of two completely different MOFs. These MOF-on-MOF structures exhibit synergistically enhanced properties compared to individual MOF subunits. Liu et al. (2020) epitaxially grew the guest MOF (ZIF-8) on the specific facets of the host MOF (MIL-125). Moreover, the growth position of ZIF-8 on MIL-125 can be selected by using MIL-125 hosts with facets exposed on the corners or side surfaces. Based on this, two different structural MIL-125@ZIF-8 heterojunctions were synthesized, enhancing their photocatalytic performance [165].

#### 4.4.2. Properties

The properties of MOFs are determined by their highly ordered pore structures, as well as the synergy between metal nodes and organic linkers [70,166]. In terms of structural characteristics, the specific surface area of MOFs can exceed 7000 m^2^ g^−1^ (e.g., NU-1501-Al, with a BET surface area reaching 7310 m^2^ g^−1^), and pore sizes can be precisely controlled within the range of 0.5–10 nm, making them suitable for gas adsorption and separation [167]. Regarding chemical properties, the stability of MOFs has been significantly enhanced [73]. For example, zirconium-based MOFs (such as UiO-66) can maintain structural integrity in aqueous solutions with a pH range of 1–12 through ligand modification, and they have good thermal stability [168]. In terms of functionalization, post-synthetic modification can enhance its catalytic activity. For instance, the high porosity of MIL-101 achieves good adsorption performance [169]. ZJU-300 exhibits superior performance in C_2_H_2_ adsorption, with an adsorption capacity reaching 3.23 mmol g^−1^ [170]. Moreover, the mechanical properties of MOFs have been optimized by introducing flexible linkers. For example, MIL-53 (Al) has a good reversible contraction rate [171].

### 4.5. Covalent Organic Frameworks

Covalent Organic Frameworks (COFs) are a class of crystalline porous polymers formed by the covalent connection of organic molecules [172,173,174]. They are characterized by their regular pore structures, highly ordered lattices, and strong designability. Typically composed of lightweight elements such as carbon, hydrogen, oxygen, nitrogen, and boron, COFs exhibit good thermal and chemical stability and can have two-dimensional or three-dimensional structural forms. The synthesis of COFs is mainly achieved through condensation reactions, solvothermal synthesis, or template synthesis. Leveraging their porosity and functionality, COFs have shown broad application prospects in multiple fields such as gas storage and separation, catalysis, energy storage, sensing, drug delivery, and environmental remediation [175,176].

#### 4.5.1. Synthesis Methods

**Figure 6:**
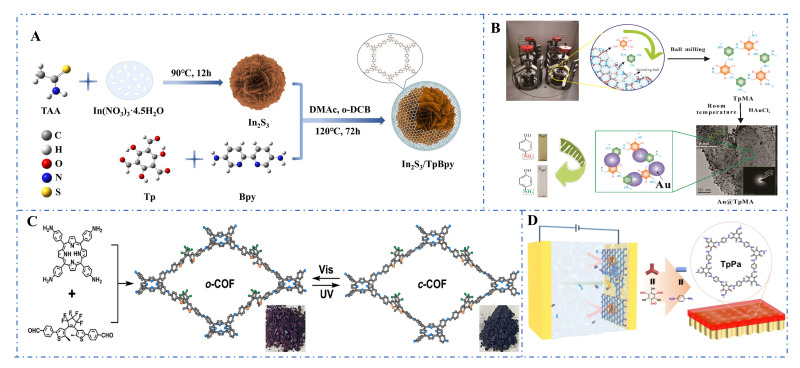
(**A**) Synthesis of In_2_S_3_/TpBpy COFs by solvothermal method [177]; (**B**) Synthesis of COFs at room temperature by ball milling [178]; (**C**) Preparation of ultrathin COF films by liquid–liquid interfacial self-assembly [179]; (**D**) Synthesis of photoresponsive COFs [180].

COFs have been continuously innovated in recent years, covering efficient preparation, structural controllability, and functional integration. Solvothermal methods enhance crystallinity by optimizing solvent systems and reaction kinetics. For example, Zhang et al. (2025) synthesized highly crystalline In_2_S_3_/TpBpy using a mixed solvent (Figure 6A), achieving a specific surface area of 640 m^2^ g^−1^ [177]. Mechanochemical synthesis has gained attention due to its solvent-free and low-energy consumption advantages. Niu et al. (2022) rapidly prepared COFs via ball milling at room temperature (Figure 6B) [178]. Interfacial polymerization techniques have also made further breakthroughs. Wang et al. (2023) reported a method for synthesizing ultrathin COF membranes via liquid–liquid interfacial self-assembly (Figure 6C). The membrane exhibited a high water flux of 91.77 kg·m^−2^·h^−1^ and provided some theoretical and technical guidance for interfacial polymerization [179]. The combination of dynamic covalent chemistry and photoresponsive technology has driven the development of smart COFs. Sum et al. (2022) achieved functional integration in the synthesis of a photoresponsive o-COF by combining porphyrin photosensitizers with diarylethene switches (Figure 6D). They also introduced an effective strategy for functionalizing COFs by integrating multiple functional building blocks, thereby further enriching the functional diversity of such materials [180].

#### 4.5.2. Properties

The properties of COFs are dominated by their crystalline porous structures connected by fully covalent bonds [172,173,181], which are characterized by high specific surface area, programmable pore sizes, and customizable functionalities. In terms of structural characteristics, COFs have large specific surface areas, and their pore sizes can be precisely controlled through monomer design, making them suitable for gas separation and catalysis. Their chemical stability is significantly enhanced. For example, COF-42, which is connected by boronic ester bonds, maintains structural integrity in a wide range of aqueous solutions with different pH values and exhibits good thermal stability [182].

In terms of functionalization, the introduction of photosensitive groups (such as porphyrins) through in situ synthesis can achieve efficient photocatalytic hydrogen production, with a hydrogen production rate of 126 mmol g^−1^ h^−1^ for TP-COF [183]. The charge transport properties have been optimized through π-π stacking, with the carrier mobility of TTF-COF increased to 1.2 cm^2^ V^−1^ s^−1^, making it suitable for organic field-effect transistors [184]. Regarding dynamic response characteristics, COF-320 with imine bonds exhibits a reversible volume expansion rate of 15% under humidity stimulation, providing a new material platform for flexible sensors [185]. The systematic regulation of these properties has propelled the applications of COFs in the fields of energy, sensing, and biomedicine.

## 5. Sensing Applications of Nanozymes

Owing to their low cost, high stability, and controllable catalytic activity, nanozymes have become an ideal substitute for natural enzymes and have shown broad application prospects in various sensing fields [45,186,187]. In colorimetric sensing, nanozymes can catalyze reactions to cause color changes in substrates, thus enabling visual detection [82,188]. In fluorescence sensing, nanozymes can combine with fluorescent substrates and produce changes in fluorescence signals through catalytic reactions, which can be used to detect various targets [65,186,189,190]. In electrochemical sensing, nanozymes can catalyze redox reactions to enhance signal output and achieve sensitive detection of target molecules [55,191]. In Raman sensing, nanozymes can serve as signal-enhancing substrates to increase Raman scattering intensity [192,193,194,195]. In chemiluminescence sensing, nanozymes can catalyze chemiluminescent reactions [196,197]. These applications demonstrate that nanozymes have significant value in the fields of biomedical detection and environmental monitoring.

### 5.1. Colorimetric Sensing

Colorimetric sensors can detect analytes through color changes, which can be read out by the naked eye or low-cost portable devices [71,160,198,199,200]. Due to their simple signal reading and rapid detection, colorimetric sensors have been widely used [201,202,203,204,205]. The instability of biological enzymes and their low catalytic activity under harsh conditions greatly hinder the detection performance of colorimetric sensors [206,207,208]. Compared with biological enzymes [77,209,210,211,212], nanozymes have good catalytic activity and high stability [213,214,215]. As a substitute for biological enzymes, nanozymes can be used to construct colorimetric sensors to improve the selectivity, sensitivity, and stability of detection [216,217,218]. The related applications are described as follows.

**Figure 7:**
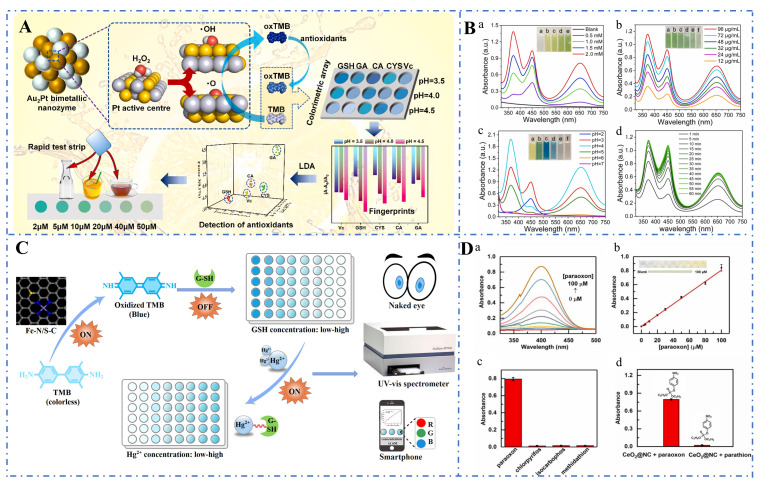
(**A**) Schematic diagram of antioxidant detection using Au_2_Pt bimetallic nanozyme arrays [219]; (**B**) Practical detection of pesticides using the inhibitory/enhancing effects of Pt NPs nanozymes (UV–vis absorbance spectra of the TMB-Pt NPs chromogenic system under different conditions for figures a, b, c, and d) [220]; (**C**) Schematic diagram of pesticide detection using the inhibitory/enhancing effects of Pt NPs nanozymes [221]; (**D**) Practical detection of organophosphorus pesticides using CeO_2_@NC nanozymes ((a) UV/Vis spectra change with various concentrations of paraoxon. (b) Standard curve for paraoxon detection. (c) Comparison of UV absorbance of different pesticides.(d) UV absorbance of paraoxon and parathion) [223].

Wu et al. (2023) constructed a colorimetric sensor array based on Au_2_Pt bimetallic nanozymes for the detection of antioxidants in food, which was successfully applied to the identification and quantitative analysis of five antioxidants, each of which exhibited a unique colorimetric response (Figure 7A) [219]. Li et al. (2022) constructed a simple and effective multi-channel colorimetric sensor array using Pt NPs as the sole nanozyme sensing receptor for the identification and detection of pesticides. Based on the differential inhibition or enhancement in the catalytic activity of Pt NPs nanozymes by pesticides, five pesticides were successfully identified (Figure 7B) [220]. Li et al. (2022) synthesized Fe-N/S-C single-atom nanozymes with oxidase-like activity using peanut shells as a template and developed a colorimetric sensor for the simultaneous detection of glutathione and Hg^2+^, which showed a wide linear range of 0.8–100 μM and 1 nM–100 μM (Figure 7C) [221]. Razavi et al. (2022) synthesized Bi_2_Fe_4_O_9_ nanoparticles by the hydrothermal method, which have high water solubility and good stability. A colorimetric sensing platform based on these nanoparticles was developed for accurate, highly sensitive, and selective detection of dopamine at the nanomolar level [222]. Gai et al. (2023) synthesized CeO_2_@NC nanozymes under mild conditions, which exhibited catalytic activity comparable to that of organophosphorus hydrolase and high stability under extreme conditions. A visual colorimetric detection method for organophosphorus pesticides was developed using these nanozymes, opening up an interesting pathway for pesticide detection (Figure 7D) [223].

### 5.2. Fluorescence Sensing

Fluorescent sensors are mainly constructed based on fluorescence enhancement (“turn-on”) or quenching (“turn-off”) mediated by the target analyte [88,142,224,225]. The emergence of nanozymes has provided a great opportunity for the development of fluorescent sensors [120,226,227,228]. In recent years, fluorescent sensors based on nanozymes have attracted extensive research interest due to their excellent performance in fluorescence signal generation and amplification [75,229,230], and they have been widely applied in many fields [231,232,233].

**Figure 8:**
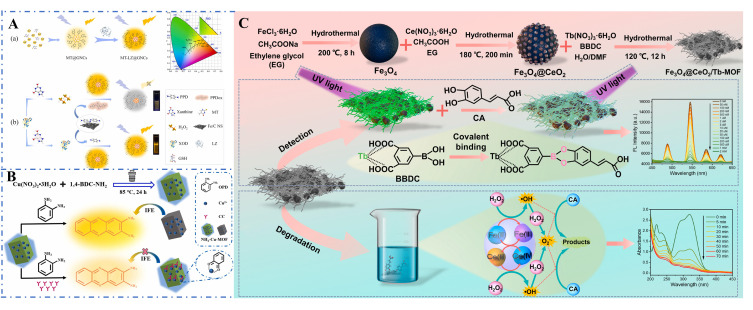
(**A**) Schematic diagram of xanthine detection using an ultrabright lysozyme-functionalized fluorescent probe ((a) Fabrication of MT-LZ@GNCs. (b) Fluorescence sensing strategy of MT-LZ@GNCs/Fe/C NS) [234]; (**B**) Schematic diagram of catechol detection by NH_2_-Cu-MOF ratiometric fluorescence [237]; (**C**) Schematic diagram of the degradation mechanism for Fe_3_O_4_@CeO_2_/Tb-MOF magnetic fluorescent nanozyme detection [238].

For example, Wang et al. (2022) synthesized a novel fluorescent nanoprobe with yellow emission based on super-bright lysozyme functionalization for the determination of xanthine, with a detection limit as low as 0.23 μmol L^−1^, enabling quantitative analysis of xanthine in the range of 0.5 to 400 μmol L^−1^ (Figure 8A) [234]. Li et al. (2022) developed a novel dual-mode fluorescent/colorimetric sensing strategy for the detection of Hg^2+^ by integrating porous cerium oxide nanorods, which exhibited a linear range of 0.08–12.5 nM and a detection limit of 0.079 nM in fluorescence assays, with the developed sensor showing high sensitivity, accuracy, and reliability [235,236]. Zhao et al. (2022) designed a ratiometric fluorescence strategy, synthesizing NH_2_-Cu-MOF with peroxidase-like activity for the detection of catechol, expanding the new application of fluorescent MOF-based nanozymes in environmental analysis (Figure 8B) [237]. Liao et al. (2024) developed a multifunctional magnetic luminescent nanozyme Fe_3_O_4_@CeO_2_/Tb-MOF, which showed excellent performance in sensitive detection and efficient degradation. The Fe_3_O_4_@CeO_2_/Tb-MOF-based fluorescent sensing had a wide linear range of 50 nM–500 μM and a low detection limit of 18.9 nM, and its magnetism allowed for recyclability, avoiding secondary pollution (Figure 8C) [238].

### 5.3. Electrochemical Sensing

The establishment of electrochemical sensors is mainly based on the changes in the output electrical signals generated from the chemical reactions between the target analytes and the immobilized electrode recognition elements [74,239,240,241]. The generation of electrical signals is usually related to the concentration of the target analytes [79,242,243], thus enabling qualitative detection and quantitative analysis of target molecules [157,244,245,246]. Electrochemical sensors have the advantages of simple operation, low cost, good stability, and high sensitivity, and have been widely used in multiple fields [21,247,248,249,250].

**Figure 9:**
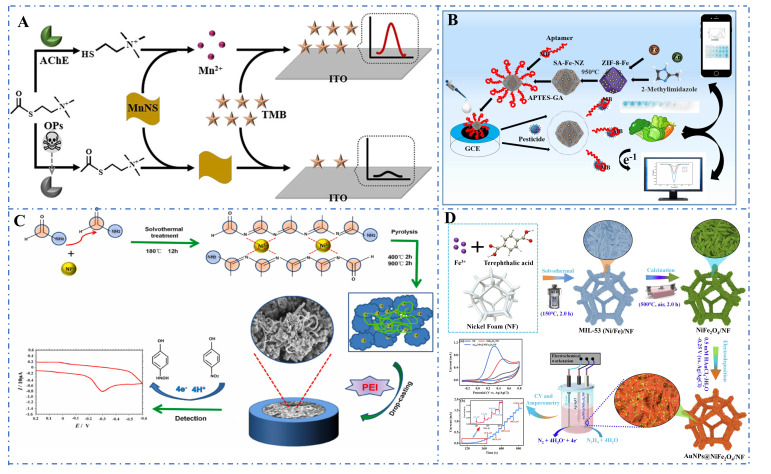
(**A**) Detection of organophosphorus pesticides using MnO_2_ nanosheet-based homogeneous electrochemical sensors [251]; (**B**) Construction of smartphone-assisted dual-mode biosensors using SA-Fe-NZ [253]; (**C**) Detection of p-nitrophenol using Ni-NCs/PEI composites [254]; (**D**) Detection of hydrazine by electrodepositing gold nanoparticles on MIL-53 (Fe, Ni) MOF-derived nanostructures [255].

Wu et al. (2021) employed nanozymes with peroxidase-like and oxidase-like properties (two-dimensional (2D) MnO_2_ nanosheets, manganese dioxide nanosheets (MnNS)) as advanced catalysts to develop a novel homogeneous electrochemical sensor for the detection of organophosphorus pesticides (OPs), using dissolved oxygen as a co-reactant to avoid interference from H_2_O_2_ and color (Figure 9A) [251]. Wei et al. (2021) developed a cobalt metal–organic framework modified carbon cloth/paper (Co-MOF/CC/Paper) hybrid button sensor as a portable, robust, and user-friendly electrochemical analytical chip for the non-enzymatic quantitative detection of glucose. A highly integrated electrochemical analytical chip with a flexible Co-MOF/CC sensing interface was successfully fabricated [252]. Wang et al. (2024) utilized the toxicity and excellent electrochemical properties of single-atom iron nanozymes (SA-Fe-NZ) to successfully construct a smartphone-assisted dual-mode biosensor, where the approach of aptamers labeled with electrochemical signaling molecules to the electrode surface caused changes in the electrochemical signal, demonstrating good detection performance (Figure 9B) [253]. He et al. (2022) developed a highly selective and sensitive p-nitrophenol (p-NP) sensor based on a composite of Ni-NCs and polyethyleneimine (PEI) (Figure 9C) [254].

Compared with bare GCE, Ni-NCs/GCE, and PEI/GCE, the Ni-NCs-PEI/GCE sensor exhibited better performance in the electrocatalytic detection of p-NP due to the synergistic effect between Ni-NCs and PEI [254]. A novel hydrazine electrochemical sensor with excellent sensing capabilities was prepared by electrodeposition of gold nanoparticles (AuNPs) on the surface of MIL-53 (Fe, Ni) MOF-derived nanostructures on a nickel foam (NF) substrate via a solvothermal method, which could perform highly sensitive hydrazine detection in tap water with good selectivity as well as reliable stability and reproducibility (Figure 9D) [255].

### 5.4. Raman Sensing

SERS has received widespread attention as an emerging and powerful analytical technique [256,257,258]. Due to its outstanding advantages, such as ultrahigh sensitivity, in situ non-invasive detection, and unique fingerprint information [259,260,261,262], it has rapidly developed in the construction of sensors [263,264,265,266]. In recent years, by utilizing nanozymes to enhance SERS performance [80,267,268], a variety of SERS-based analytical platforms have been successfully established for the sensitive detection of multiple target molecules [269,270,271].

**Figure 10:**
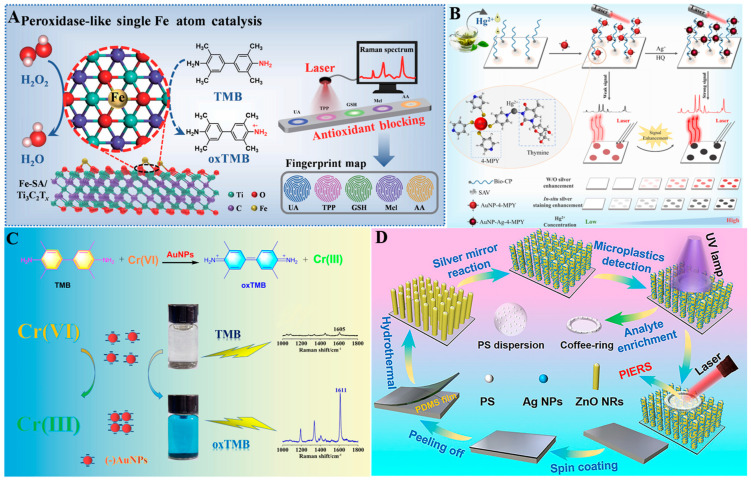
(**A**) Detection of antioxidants using Fe-SA/Ti_3_C_2_Tx nanozyme-enhanced Raman spectroscopy [272]; (**B**) Dual-mode paper sensor based on AuNPs/4-MPy for Hg^2+^ detection [273]; (**C**) Detection of Cr (VI) by dual-functional AuNPs-catalyzed oxidation of TMB [274]; (**D**) Detection of microplastics using biomimetic Ag/ZnO nanorod arrays [276].

Xi et al. (2023) developed iron single atoms (Fe-SA/Ti_3_C_2_Tx) with intrinsic peroxidase-like activity loaded on Ti_3_C_2_Tx (Figure 10A) and further constructed a sensitive Raman sensor array for the detection of five antioxidants, achieving satisfactory signal amplification performance. By utilizing the blocking effect of radical reactions and its highly recognizable catalytic characteristics, it can simultaneously identify antioxidants, including ascorbic acid, uric acid, glutathione, melatonin, and tea polyphenols [272]. Chen et al. (2025) synthesized a dual-mode paper sensor based on AuNPs and 4-mercaptopyridine for rapid and ultrasensitive detection of Hg^2+^ in tea. The sensor combined SERS technology with a silver shell grown in situ on the surface of AuNPs to significantly enhance signal intensity (Figure 10B). The detection limit of SERS was 0.48 pM, which is 500 times lower than that of traditional methods [273]. Xu et al. (2022) designed a novel SERS detection method for Cr (VI) detection through the catalytic oxidation of TMB by bifunctional (−)AuNPs (Figure 10C). The detection exhibited excellent selectivity with a detection limit as low as 0.4 nM [274]. Shaikh et al. (2023) synthesized silver nanostructures (Ag@Ch) loaded on a corrugated chitosan matrix for highly sensitive SERS detection of methylene blue, crystal violet, and p-nitrophenol [275]. Zhu et al. (2023) prepared Ag/ZnO nanorods on a PDMS film by mimicking the nanorod array on the surface of a dragonfly wing (Figure 10D). By combining semiconductor photoinduction and bionic nanostructures, the visualization and quantitative analysis of plastic microparticles were achieved, with the generated PIERS enhancement ratio being 11.3 times higher than that of normal SERS [276].

### 5.5. Chemiluminescence Sensing

Chemiluminescent sensors, as a promising analytical tool, are constructed based on light emission generated from chemical reactions [277,278,279]. They have the advantages of convenient operation, rapid response, simple equipment, low limit of detection (LOD), and wide linear range, and have been widely used for the detection of various targets [277,280,281]. The emergence of nanozymes also provides a promising strategy for the design and development of chemiluminescent biosensors.

**Figure 11:**
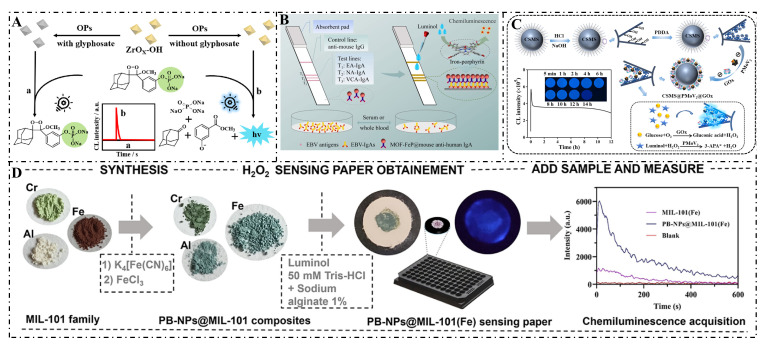
(**A**) Detection of glyphosate using ZrOX-OH nanozyme-catalyzed dephosphorylation of AMPPD [282]; (**B**) Detection of EB virus antibodies using MOF-FeP [283]; (**C**) Enhanced detection of dopamine by CoMoO_4_ nanorods in the chemiluminescence system of luminol/H_2_O_2_ [286]; (**D**) Detection of H_2_O_2_ using Prussian blue/MIL-101 nanozyme-based paper sensor [285].

Chang et al. (2023) synthesized a zirconium hydroxide nanozyme (ZrOX-OH) with phosphatase-like activity, achieving direct and specific chemiluminescent detection of glyphosate. The nanozyme was prepared by simple alkaline solution treatment of UIO-66 and could catalyze the dephosphorylation of the substrate AMPPD to produce a strong chemiluminescent signal (Figure 11A) [282]. Wang et al. (2023) synthesized a chemiluminescent test paper based on an iron porphyrin single-atom nanozyme (MOF-FeP) for rapid and highly sensitive detection of Epstein–Barr virus antibodies related to nasopharyngeal cancer. The nanozyme, by mimicking the active sites of natural peroxidase, exhibited excellent peroxidase-like activity and could catalyze the chemiluminescence of the luminol substrate (Figure 11B) [283]. Dong et al. (2024) synthesized bimetallic CoMoO_4_ nanorods, which were successfully prepared with high peroxidase activity through hydrothermal and subsequent calcination processes. These nanorods were first used as chemiluminescent catalysts, enhancing the CL intensity of the luminol/hydrogen peroxide system by nearly 750 times. A sensitive and rapid detection platform for dopamine was established based on the quenching effect of dopamine on chemiluminescent signals [284].

And Martínez-Pérez-Cejuela et al. (2024) developed a chemiluminescent sensing paper based on a Prussian blue/metal–organic framework MIL-101 nanozyme for rapid detection of hydrogen peroxide (H_2_O_2_) (Figure 11D). Prussian blue nanoparticles (PB-NPs) were first grown in situ on the MIL-101 (Fe) structure to form a PB-NPs@MIL-101 (Fe) composite. This composite was used to prepare a one-step H_2_O_2_ detection sensing paper, capable of detecting H_2_O_2_ down to 8.2 μM with good reproducibility and storage stability [285]. Jia et al. (2023) developed a novel nanochannel-confined biomimetic nanozyme/bioenzyme cascade reaction system (CSMS@PMoV_2_@GOx) for generating persistent and intense chemiluminescence. The system co-immobilized polyoxometalates (PMoV_2_) and glucose oxidase (GOx) in the nanochannels of core-shell mesoporous silica microspheres (CSMS), enabling the production of hydrogen peroxide (H_2_O_2_) from glucose and luminol reactions, which in turn triggered chemiluminescent emission (Figure 11C) [286].

## 6. Practical Implementation Challenges

The practical application of nanozymes in sensing and analysis is constrained by various methodological limitations. First, the catalytic mechanisms of nanozymes have not been fully elucidated, especially the dynamic behavior of their active sites and the mechanisms of substrate binding, which lack a unified theoretical explanation. For example, the peroxidase-like activity of noble metal nanozymes (such as Au and Pt) is usually attributed to the electron transfer capability of surface metal atoms [287], but the differences in their catalytic pathways compared to natural enzymes (such as horseradish peroxidase) remain unclear [267,288]. The redox activity of metal oxide nanozymes (such as Fe_3_O_4_ and CeO_2_) is related to their multivalent characteristics, but how to optimize catalytic efficiency through precise regulation of oxygen vacancies or surface functional groups remains a technical challenge [112,289]. In addition, the enzyme-like activity mechanisms of carbon-based nanozymes (such as graphene quantum dots) are highly dependent on their surface defects and heteroatom doping, but existing characterization methods (such as in situ X-ray photoelectron spectroscopy) are unable to track the microstructural changes during the catalytic process in real time, limiting the development of rational design strategies [130,290].

Second, the selectivity and specificity of nanozymes remain technical bottlenecks. Compared with natural enzymes, nanozymes exhibit weaker substrate recognition capabilities and are susceptible to interference from coexisting substances in complex matrices. Notably, food extracts containing fats, proteins, and reducing agents (e.g., ascorbic acid) profoundly suppress nanozyme activity through three key mechanisms: (1) hydrophobic fats form bio-coronas that block active sites; (2) proteins adsorb onto surfaces via electrostatic interactions, reducing catalytic accessibility; (3) reducing agents competitively consume reactive oxygen species (ROS), causing false-negative signals. Although surface modification (e.g., ligand functionalization) may partially improve selectivity, this process risks masking active sites or introducing non-specific adsorption [88,145].

Finally, the long-term stability and large-scale production of nanozymes are still unresolved problems. Although some nanozymes (such as MOFs and COFs) are stable in mild environments under laboratory conditions, in practical applications, extreme pH, high temperature, or high ionic strength may lead to structural collapse or deactivation of active sites. For example, Zr-MOFs are prone to ligand hydrolysis under strongly acidic conditions, limiting their application in gastric juice detection. In addition, the high cost and complex synthesis process of noble metal-based nanozymes hinder their large-scale commercialization, while the low activity of carbon-based or metal oxide nanozymes needs to be improved through complex post-treatment (such as doping and compositing), further increasing production difficulties [291,292].

## 7. Outlook

The future development of nanozymes in the field of sensing and analysis will revolve around material innovation, mechanistic exploration, and application expansion. First, designing new nanozyme materials through interdisciplinary approaches (such as computational chemistry and machine learning) is expected to break through the current limitations of catalytic activity and selectivity. For example, using high-throughput screening techniques combined with molecular dynamics simulations can accurately predict nanostructures with specific enzyme activities, thereby accelerating the development of high-performance nanozymes. Second, in-depth elucidation of the catalytic mechanisms of nanozymes is a key research direction. Revealing the dynamic structural changes during the catalytic process through in situ characterization techniques (such as in situ X-ray absorption spectroscopy and cryo-electron microscopy) will provide theoretical support for optimizing nanozyme performance.

In terms of applications, the integration of multifunctional sensing systems is a future trend. For example, combining nanozymes with microfluidic technology to develop “lab-on-a-chip” platforms can enable parallel detection and real-time monitoring of multiple targets. In addition, the integration of nanozymes with smart materials (such as photo-responsive and magnetically responsive materials) can build environmentally adaptive sensing systems, enhancing detection reliability in complex scenarios. In the fields of environment and health, nanozyme sensing technology is expected to further expand into in situ remediation of pollutants and early disease diagnosis. For example, developing self-cleaning nanozyme sensors can simultaneously detect and degrade organic pollutants in water, while wearable sensors based on nanozymes may provide continuous monitoring solutions for chronic disease management.

Finally, promoting the green synthesis and sustainable application of nanozymes is crucial. Preparing low-cost nanozymes through biotemplating or using waste-derived carbon materials can reduce environmental burdens and improve resource utilization. At the same time, establishing international unified performance evaluation standards and toxicity assessment systems will accelerate the transition of nanozymes from the laboratory to industrialization. With continuous technological breakthroughs, nanozymes are expected to become a core component of the next generation of intelligent sensing systems, providing more efficient and precise solutions for food safety, medical diagnosis, and environmental protection. Moreover, large-scale deployment of nanozymes raises concerns regarding potential environmental release and ecotoxicity. As nanozymes are increasingly applied in food safety detection and other fields, their production, use, and disposal may lead to the release of nanoparticles into the environment. Therefore, stringent regulatory frameworks and ecological risk assessment systems should be established to ensure that large-scale deployment of nanozymes does not pose unacceptable risks to ecosystems and human health.

## Figures and Tables

**Table 1:** Comparison between natural enzymes [56,57,58,59] and nanozymes [60,61,62].

Characteristics	Natural Enzymes	Nanozymes
High catalytic activity	✓	✓
High Substrate Selectivity	✓	×
Good Biocompatibility	✓	✓
Broad Biocatalytic Scope	✓	✓
Genetic/Protein Engineering	✓	×
High Cost	×	✓
Limited Stability	×	✓
Difficult Long-Term Storage	×	✓
Recyclability	×	✓

## Data Availability

No new data were created or analyzed in this study. Data sharing is not applicable to this article.
